# Juvenile Plasma Factors Improve Organ Function and Survival following Injury by Promoting Antioxidant Response

**DOI:** 10.14336/AD.2021.0830

**Published:** 2022-04-01

**Authors:** Xiaogang Chu, Kumar Subramani, Bobby Thomas, Alvin V Terry, Sadanand Fulzele, Raghavan Pillai Raju

**Affiliations:** ^1^Department of Pharmacology and Toxicology, Medical College of Georgia, Augusta, GA 30912, USA.; ^2^Departments of Pediatrics, Neuroscience and Drug Discovery, Darby Children’s Research Institute, Medical University of South Carolina, Charleston, SC 29425, USA.; ^3^Department of Medicine, Medical College of Georgia, Augusta, GA 30912, USA.

**Keywords:** aging, exosomes, extracellular vesicles, shock, juvenile factors

## Abstract

Studies have shown that factors in the blood of young organisms can rejuvenate the old ones. Studies using heterochronic parabiosis models further reinforced the hypothesis that juvenile factors can rejuvenate aged systems. We sought to determine the effect of juvenile plasma-derived factors on the outcome following hemorrhagic shock injury in aged mice. We discovered that pre-pubertal (young) mice subjected to hemorrhagic shock survived for a prolonged period, in the absence of fluid resuscitation, compared to mature or aged mice. To further understand the mechanism of maturational dependence of injury resolution, extracellular vesicles isolated from the plasma of young mice were administered to aged mice subjected to hemorrhagic shock. The extracellular vesicle treatment prolonged life in the aged mice. The treatment resulted in reduced oxidative stress in the liver and in the circulation, along with an enhanced expression of the nuclear factor erythroid factor 2-related factor 2 (Nrf2) and its target genes, and a reduction in the expression of the transcription factor BTB and CNC homology 1 (Bach1). We propose that plasma factors in the juvenile mice have a reparative effect in the aged mice in injury resolution by modulating the Nrf2/Bach1 axis in the antioxidant response pathway.

Aging systems reduce agility of the organism and contribute to an increased propensity for injuries [1, 2]. Furthermore, the age-associated changes in the cellular response to injury impair recovery [3-6]. Our laboratory and others have demonstrated a declined organ function following hemorrhagic shock injury (HI) which is exacerbated with aging [2, 5-8]. Oxidative stress has been reported to be one of the critical pathological hallmarks of HI [9, 10]. The inter-relationship between oxidative stress and cellular energetics triggers a vicious cycle post-injury, as an excessive production of oxidants results in impaired cellular functions leading to organ dysfunction and mortality. The transcription factor nuclear factor erythroid 2-related factor 2 (Nrf2) is a "master regulator" of the antioxidant responses and plays a key role in maintaining redox homeostasis [11]. Nrf2 modulates the expression of hundreds of genes, that regulate antioxidants, cytoprotection, anti-inflammatory processes, tissue remodeling and fibrosis, carcinogenesis and metastasis [12, 13]. Thus, the dysregulation of the Nrf2 signaling network of critical gene products is a likely cause for the perpetuation of oxidative stress resulting in the disease pathology.

While exogenous agents that modulate injury response pathways are being extensively investigated, very little is known about endogenous factors that can restore homeostasis following injury. Transfer of young blood to old animals by heterochronic parabiosis is known to have protective effects in injury as well as in organismal aging [14-16]. However, the identity or the mechanism of maturation-dependent factors in the blood that have reparative effects remain to be determined. Studies have demonstrated that transfer of blood from young animals to aged ones reduced infarct size in stroke [17], reversed age-related cardiac hypertrophy [18], and rejuvenated aged progenitor cells [19]. Stem cells and stem-cell derived extracellular vesicles (EVs) were investigated in several laboratories as therapeutic agents for different disease conditions [20-22]. The EVs derived from bodily fluids, including blood plasma, have been effectively used in experimental models of human diseases [23, 24]. In this study, we hypothesized that juvenile plasma-derived EVs can potentiate metabolic networks converging on cellular energetics, attenuate oxidative stress, and improve outcomes following injury in the aged mice. We tested this hypothesis by treating aged mice subjected to HI with EVs isolated from young mice (Y-EVs).

## MATERIALS AND METHODS

### Animals

**Figure 1.**
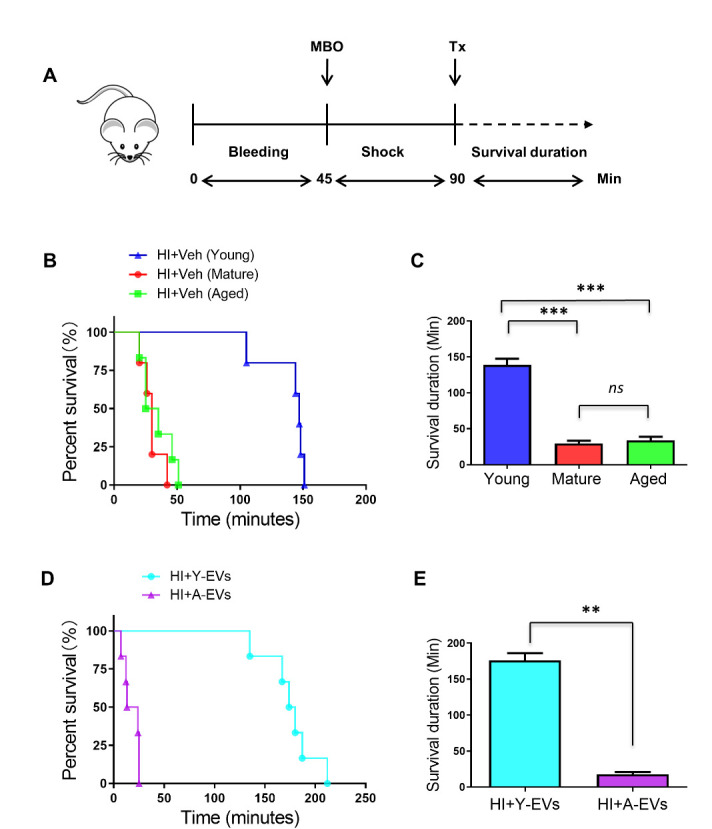
Effect of juvenile plasma factors on survival following HI. (A) Schematic representation of the experimental design for the hemorrhagic shock injury (HI): model for survival studies (no fluid resuscitation). MBO=maximum bleed out. Tx = treatment with Veh or EVs. (B) Kaplan-Meier survival curves for young, mature and aged mice subjected to HI. **indicates p<0.01; Veh=vehicle (C) Bar diagram represents mean duration of survival of the animals represented in panel B; mean ± SEM; ***indicates p<0.001. Survival duration is time from treatment (Tx). (D) Kaplan-Meier survival curves for aged mice subjected to HI. Y-EVs or A-EVs were administered after the shock period. EVs were isolated from the plasma of young (Y-EVs) or aged (Y-EVs) mice. (E) Bar diagram represents mean duration of survival of the animals represented in panel D; mean ± SEM; **indicates p<0.01. Survival duration is time from treatment (Tx).

**Figure 2.**
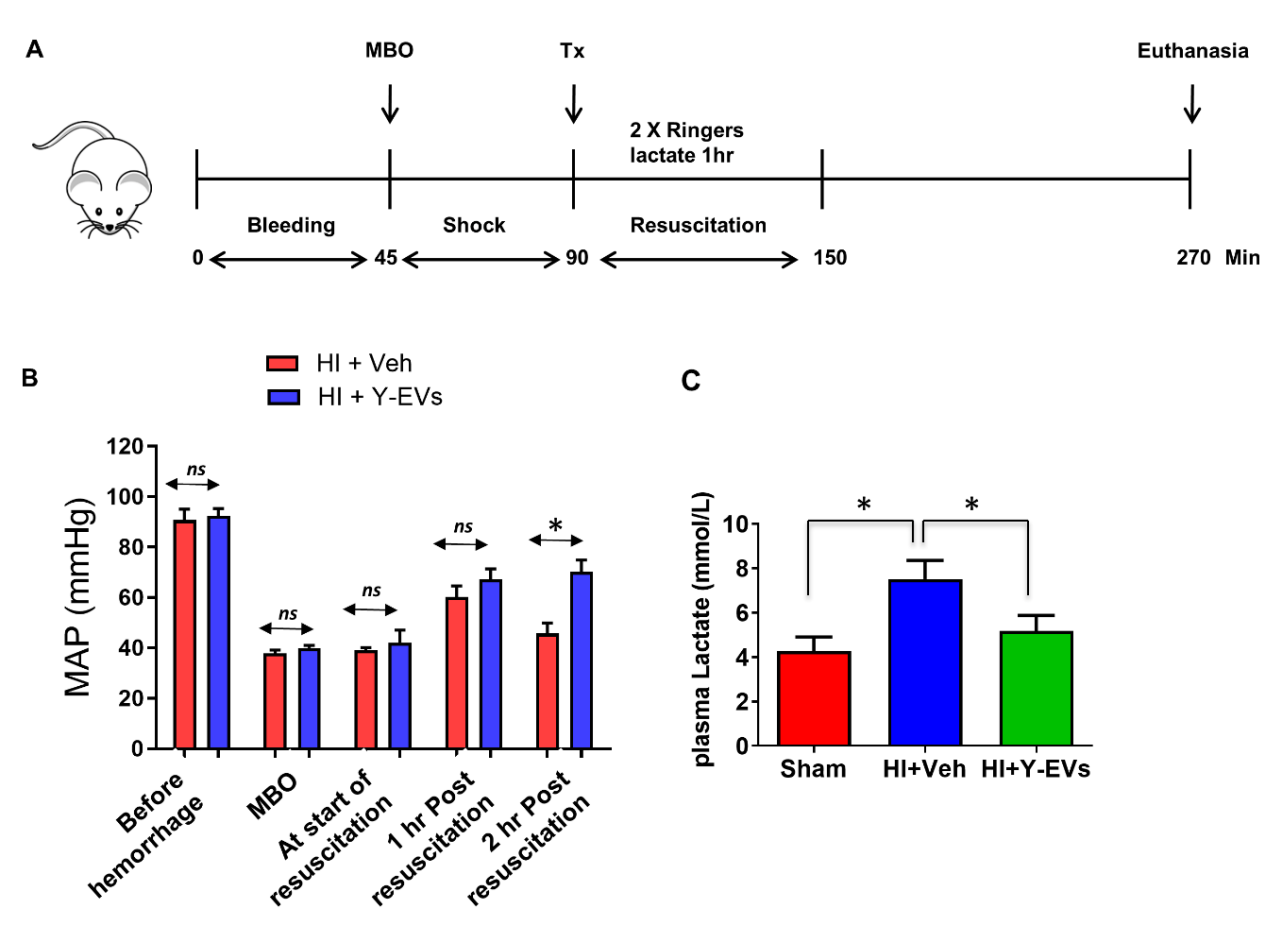
MAP and plasma lactate improved with Y-EVs treatment following HI. (A) Schematic representation of the experimental design for HI: model for organ function studies (with fluid resuscitation). (B) Mean arterial pressure (MAP) following HI and treatment with Y-EVs measured before hemorrhage, at maximum bleed out (MBO), at start of resuscitation, and at 1 and 2 h post resuscitation. mean ± SEM. *p < 0.05; ns = not statistically significant. Two-way ANOVA and Tukey’s multiple comparisons post hoc tests were used. (C) Plasma lactate levels. Groups: Sham, HI+Veh and HI+Y-EVs; mean ± SEM, *indicates p<0.05.

Young (5-6-week-old) and mature (3-4-month-old) male C57BL/6 mice used were obtained from Jackson laboratories and aged (23-26-month-old) C57BL/6 mice were from the National Institute of Aging rodent colony. Animal studies were conducted as per the protocol approved by the Institutional Animal Use and Care committee at the Augusta University. Hemorrhagic shock was induced as described before [25]. Briefly 60% blood volume was removed in 45 minutes through femoral artery, to induce hypovolemic shock (Mean arterial pressure: 30-35 mm Hg) and this MAP was maintained for another 45 minutes (shock period). Sham animals were not subjected to bleeding or fluid resuscitation, but they were subjected to laparotomy and groin incisions. For survival studies, EVs or vehicle was given immediately after the shock period (Fig. 1A). Each group for this study had 5-6 animals. For organ function studies, the animals were resuscitated following shock period for 1 hour, with Ringers Lactate (twice the shed blood volume), observed for another two hours and sacrificed to remove tissues for mitochondrial function assessment (Fig. 2A). Each group for this study had 6 animals. In this latter group, EVs or vehicle was administered as a single dose (50 µl; 2 mg/Kg body weight) at 10 minutes after the start of fluid resuscitation.

### Preparation of EVs

The plasma EVs-enriched fractions were prepared as follows and as described in Ref. [26]. Briefly, plasma was diluted into 500 µL PBS followed by centrifugation at 3000 RPM for 20 min to remove cell debris. The supernatant was collected and again centrifuged at 8000 RPM for 30 min to remove the remaining cell debris (1-3). The supernatant was collected and then Total Exosome Isolation Reagent (Life Technologies, Carlsbad, CA, USA) was used to isolate EVs as per manufacturer protocol. This protocol involved initial precipitation followed by centrifugation. After centrifugation, pellets were dissolved in phosphate-buffered saline (PBS) as EVs enriched fractions. EV concentrations were normalized by total protein level.

### Mitochondrial respiration

The Seahorse XFp Analyzer (Seahorse Biosciences, North Billerica, MA) was used according to the manufacturer’s protocol to measure oxygen consumption rate (OCR). Splenocytes or MEF cells were plated in XFp base medium, minimal DMEM (Seahorse Biosciences, North Billerica, MA) supplemented with 1mM pyruvate, 2mM glutamine, 10mM glucose (Sigma) at a density of 3 X 10^5^ cells per well in specialized XFp miniplates pretreated with Cell Tak (Fisher). Plates were spun for 1 minute at 200 x g to ensure cell adhesion, and then incubated for 30 minutes at 37 °C prior to loading into the Seahorse analyzer. The following mitochondrial inhibitors were sequentially injected: oligomycin (1 μM), carbonycyanide p-(trifluoromethoxy) phenylhydrazone (FCCP) (0.3 μM), antimycin A and rotenone (1 μM) and OCR measured. OCR values of the cells from animals in HI group were normalized to that of the sham-operated animals. Samples from 6 sham animals and 4 animals each in each HI and treatment groups were used.

*Liver mitochondria:* Mice liver mitochondria were extracted using tissue mitochondria isolation kit (#k288-50, BioVision, Milpitas, CA). Samples were from 4 sham animals and 3 animals each in each HI and treatment groups. Isolated liver mitochondria were diluted to 4µg/25µl in cold MAS buffer + substrate (pyruvate/malate) and plated onto 8-well XFp plates for OCR measurements. The plate was centrifuged at 2000g for 20 min at 4°C to attach the mitochondria. After centrifugation, an additional 155µl of MAS buffer + substrate was added to the wells. Mitochondria were checked under the microscope to ensure a homogeneous mitochondrial monolayer in each well and incubated at 37°C for 10 min. The plates were further equilibrated for 8 min before the measurement of basal respiration. The final concentrations of compounds after injections were as follows: 4 mM ADP, 3 µM oligomycin, 4 µM FCCP, and 4 µM antimycin A. Respiratory state was calculated by the Seahorse XF software package

### JC-1 assay for mitochondrial membrane potential

The experiment was performed using Isolated Mitochondria Staining kit (Sigma Chemical Co., St. Louis, MO). Samples from 4 animals were used in each group. Briefly, 5μg sample of isolated mitochondria (BioVision, Milpitas, CA) was stained with a 0.2 μg/mL JC-1 solution. For a control, valinomycin, an ionophore, was added to an identical 5μg sample of isolated mitochondria to a final concentration of 0.5μg/ml. Four animals were used in each group. This sample was placed on ice for 10 minutes to allow complete dissipation of the membrane potential, and then stained with a 0.2 μg/mL JC-1 solution and assayed in parallel. Samples were read over a period of 30 minutes using a BIOTEK (Synergy HT) fluorescence plate reader (excitation wavelength = 485 nm, emission wavelength = 590 nm). Results are expressed as a percentage of the valinomycin baseline control fluorescence.

### Cell Culture

WT and NRF2^-/-^ MEF cells were maintained as sub confluent monolayers in Dulbecco's Modified Eagle's medium (Gibco, Waltham, MA) supplemented with 10% fetal bovine serum (HyClone, Logan, UT) and 100 units/ml penicillin plus 100 μg/ml streptomycin (Invitrogen, Waltham, MA) at 37°C in 5% CO2. Cells were treated with 100 μg/ml EVs or media for the durations indicated.

### Immunoblotting Analysis

Immunoblotting procedures were performed as we described previously [25]. Briefly, tissues or cells were homogenized in the lysis buffer. Lysates were clarified at 12,000 g for 10 min at 4°C, and protein concentrations were determined by the Bradford protein assay (Bio-Rad Laboratories, Hercules, CA). Equal amounts of protein were loaded onto 8-12% SDS-PAGE, transferred onto polyvinylidene difluoride membranes, probed with the indicated primary antibody and the appropriate secondary antibody conjugated with horseradish peroxidase (Cell Signaling, Danvers, MA), and the immune complexes were detected by standard methods. Samples from 5-6 animals were used for this study. 2-3 technical replicates were used in each experiment. 6 replicates were used for *in vitro* experiments except for Figure 8B where 4 replicates were used. The antibodies used in this study are listed in Supplementary Table 1.

### Real-Time Polymerase Chain Reaction

Total RNA was isolated using TRIZOL reagent (Thermo Fisher Scientific, Waltham, MA) and cDNA was synthesized using ImProm-II™ Reverse Transcription System (Promega, Madison, WI). Realtime PCR was performed using standard protocols. Samples from 5-6 animals were used for PCR analysis. 2-3 technical replicates in each experiment. 6-9 replicates were used for *in vitro* experiments. The sequences of the primers used in this study are listed in Supplemetary Table 2.

### Lactate assay

The plasma was separated by centrifugation (2,000?g, 10?min) and stored at -80?°C until assayed for lactate levels. Samples from 5-6 animals were used. Lactate levels were measured using a Lactate Assay Kit (Sigma, St. Louis, MO) according to the manufacturer's protocol.

### MDA and H_2_O_2_ assay

Plasma malondialdehyde (MDA) was determined using the TBARS (Thiobarbituric Acid Reactive Substances Assay) Kit (Cayman Chemical, Ann Arbor, MI) according to the manufacturer's protocol. Samples from 5 animals/group were used. H_2_O_2_ assay was carried out using the Amplex Red Assay from Invitrogen (Thermofisher, MA) as per the manufacturer protocols.

### NRF2 transcription factor assay

Mice liver nuclei were extracted using tissue nuclear extraction kit (Active motif, Carlsbad, CA) according to the manufacturer's protocol. Samples from 5 animals/group were used. Nrf2 transcription factor DNA binding activity were detected using Nrf2 transcriptional factor assay kit (#600590, Cayman Chemical, Ann Arbor, Michigan).

### Statistics

Survival analysis was performed by Kaplan-Meier plot and significance between survival curves was determined using Graphpad Prism 9 (GraphPad Software, San Diego, CA). All statistical analysis were done using Graphpad Prism software. Data normality was tested by Shapiro-Wilk normality test. One-way ANOVA with Tukey's post hoc correction was used for normal data. Studies with more than one independent variable were analyzed by two-way ANOVA with post hoc correction. Non-parametric test was done for data that did not pass the normality test. Two group comparisons were done by Mann-Whitney t-test.

## RESULTS

The severe blood loss resulting in decompensated hemorrhagic shock is fatal unless the animals receive prompt fluid resuscitation or agents that can prolong life. These animals do not survive more than an hour following the shock period without intervention [25]. In this study, when mature or aged mice were subjected to HI and were not resuscitated with fluid, the mean duration of survival observed was 30 and 34 minutes respectively (Fig. 1A-B), and the survival durations were not significantly different. However, when pre-pubertal (young) mice were subjected to hemorrhagic shock, in the absence of fluid resuscitation, these animals survived for a significantly prolonged period of time (mean=139 minutes) compared to mature or aged animals (Fig. 1-B). The results showed that young mice were more resilient than mature or aged mice to the severe whole-body insult of hemorrhagic shock. This experiment led us to test whether plasma factors in young mice could prolong survival in the aged mice following HI.

We isolated extracellular vesicles (EVs) from the plasma of young mice (Y-EVs) and intravenously administered them to aged mice after HI, without resuscitating the mice with fluid. The aged animals that received Y-EVs survived for 176 minutes (mean value), compared to vehicle (veh)-treated mice that survived for 34 minutes (Fig. 1B-E). To test whether the improved survival was due to non-specific effect of EVs, another group of aged mice were treated with EVs isolated from aged mice. The aged mice that received EVs from the matching age group (A-EVs) showed a survival rate comparable to the animals that did not receive EVs (Fig. 1D-E) demonstrating that EVs in the plasma of young mice, but not the EVs in the plasma of aged mice, have the reparative capacity in terms of prolonging survival after HI. Y-EVs also improved the mean arterial pressure (MAP) of aged mice following the HI procedure (Fig. 2A-B). As shown in Figure 2C, plasma lactate, an indicator of hypoxemia and OXPHOS-glycolytic switch, was significantly increased in animals subjected to HI compared to sham operated animals, but was restored in animals that received Y-EVs after HI.

**Figure 3.**
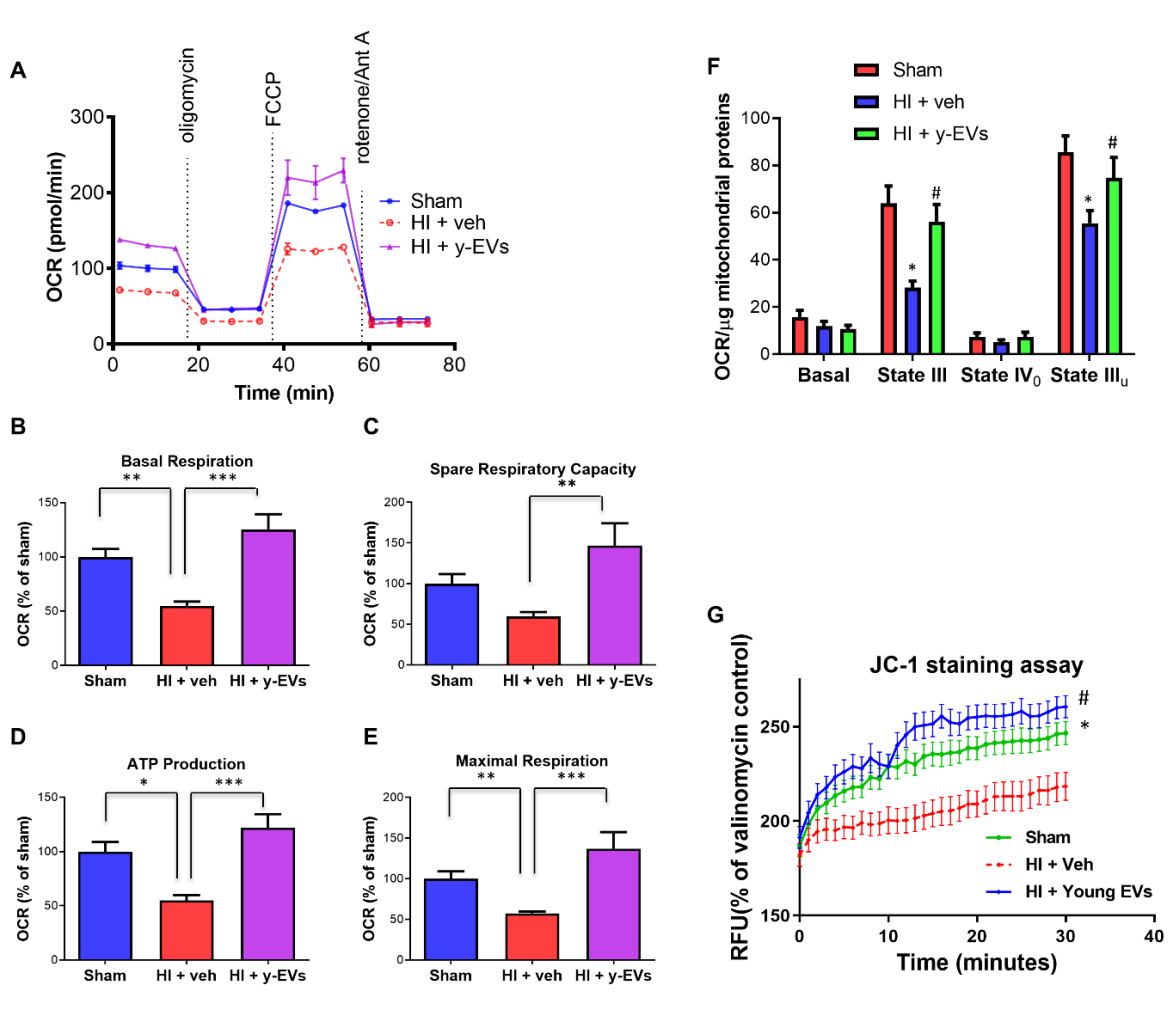
Mitochondrial function improved with Y-EVs treatment following HI. (A-E) Mitostress test on splenocytes. Splenocytes were isolated from aged mice in the following groups: sham, HI+Veh and HI+Y-EVs. Mitostress test was performed by sequential addition of oligomycin (complex V inhibitor), FCCP (mitochondrial membrane depolarization), rotenone (complex I inhibitor) and antimycin (Anti A- complex III inhibitor) in Seahorse analyzer. *= p<0.05, **=p<0.01 and ***=p<0.001. (F) Liver mitochondrial function assessment. Respiration of isolated mitochondria was measured using Seahorse analyser as described in Methods section. Groups: Sham, HI+Veh and HI+Y-EVs. *= p<0.05 compared to sham, # = p<0.05 compared to HI+Veh. (G) Liver mitochondrial permeability. Mitochondria were isolated from mouse liver as described in Methods section. Groups: Sham, HI+Veh and HI+Y-EVs. *= p<0.05 compared to sham at time=30mins # = p<0.05 compared to HI+Veh at time=30 mins (Two-way ANOVA and Tukey’s multiple comparisons post hoc tests were used). RFU - Relative Fluorescence units.

Next, considering the significance of cellular energetics in organ function maintenance, we tested whether Y-EVs treatment had any effect on mitochondrial respiration in the mice after HI. We isolated splenocytes from mice in experimental and control groups and evaluated mitochondrial respiration using a Seahorse analyzer. While the mito-stress test showed a significant decrease in the oxygen consumption rate (OCR) in aged mice following HI, Y-EVs treatment restored the OCR in the splenocytes (Fig. 3A-E). The declined basal respiration, spare respiratory capacity, ATP production, as well as maximal respiration after HI were restored in Y-EVs-treated aged mice (Fig. 3B-E). In order to test whether a similar effect on mitochondrial function was present in other tissues, we isolated mitochondria from the liver of aged sham mice, mice subjected to HI and mice subjected to HI, but treated with Y-EVs. The liver is a critical organ affected in hemorrhagic shock [27]. HI significantly decreased state III respiration and Y-EVs treatment attenuated the decrease (Fig. 3F). Consistent with these results, using mitochondria isolated from the liver, we observed that Y-EVs improved mitochondria membrane potential following HI (Fig. 3G). The declined mitochondrial function observed in various tissue compartments following HI is consistent with previous reports from our laboratory and others demonstrating mitochondrial dysfunction following HI [28] [29] [30]. However, the improvement in mitochondrial respiration following the Y-EVs treatment demonstrates the effect of Y-EVs in maintaining cellular homeostasis following HI.

**Figure 4.**
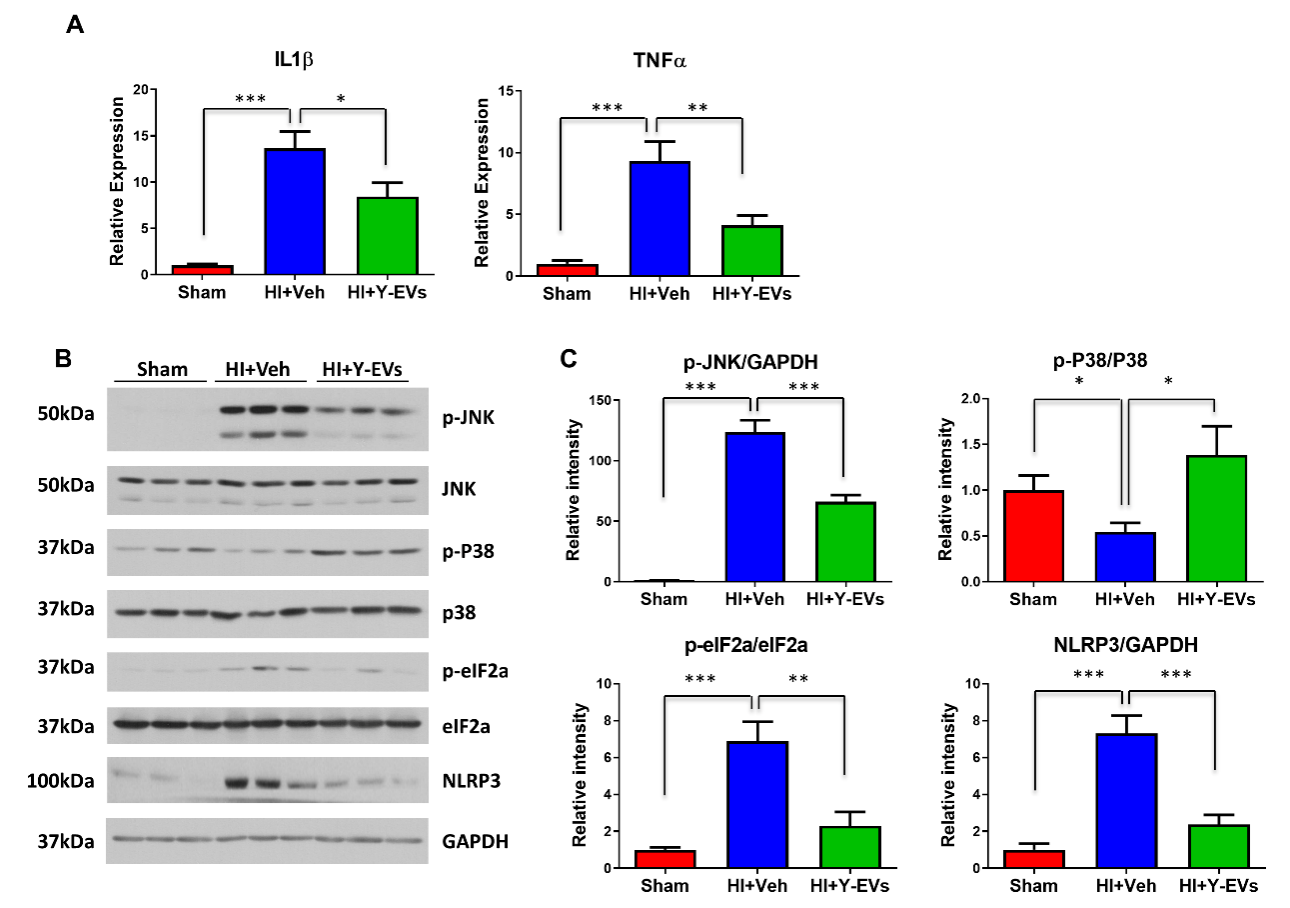
Effect of Y-EVs on liver inflammation following HI. (A) SYBR green real-time PCR amplification of IL1β and TNFα from the liver tissues. Groups: sham, HI+Veh and HI+Y-EVs; mean ± SEM; *= p<0.05, **=p<0.01 and ***=p<0.001. (B) Representative Immunoblotting of p-JNK, JNK, p-P38, P38, p-eIF2a, eIF2a and NLRP3 using liver tissues. Groups: sham, HI+Veh and HI+Y-EVs. GAPDH was used as the loading control. the blots shown are representative of the replicates. (C) p-JNK/GAPDH, p-P38/P38, p-eIF2a/eIF2a and NLRP3/GAPDH ratios were quantified using Image J software (NIH), *= p<0.05, **=p<0.01 and ***=p<0.001.

Next, we checked the gene and protein expression of molecular mediators of injury in the liver by real-time PCR and Western blot. As shown in Figure 4A, the mRNA expression of inflammatory cytokine genes IL1β and TNFα was significantly elevated in the liver of mice subjected to HI, and the increase was attenuated in Y-EVs treated mice compared to the mice in Veh treated group. Consistent with the reduction of inflammatory and injury markers in Y-EVs treatment, phosphorylated JNK, phosphorylated eIF2α, as well as NLRP3 levels were reduced while the level of phosphorylated P38 in Y-EVs treated animals was increased as compared to the controls indicating injury resolution following Y-EVs treatment (Fig. 4B-C). The results indicate significant reduction in cell stress (p-JNK and Eif2a) and inflammation (NLRP3) following injury in animals treated with Y-EVs, thus demonstrating a therapeutic effect.

**Figure 5.**
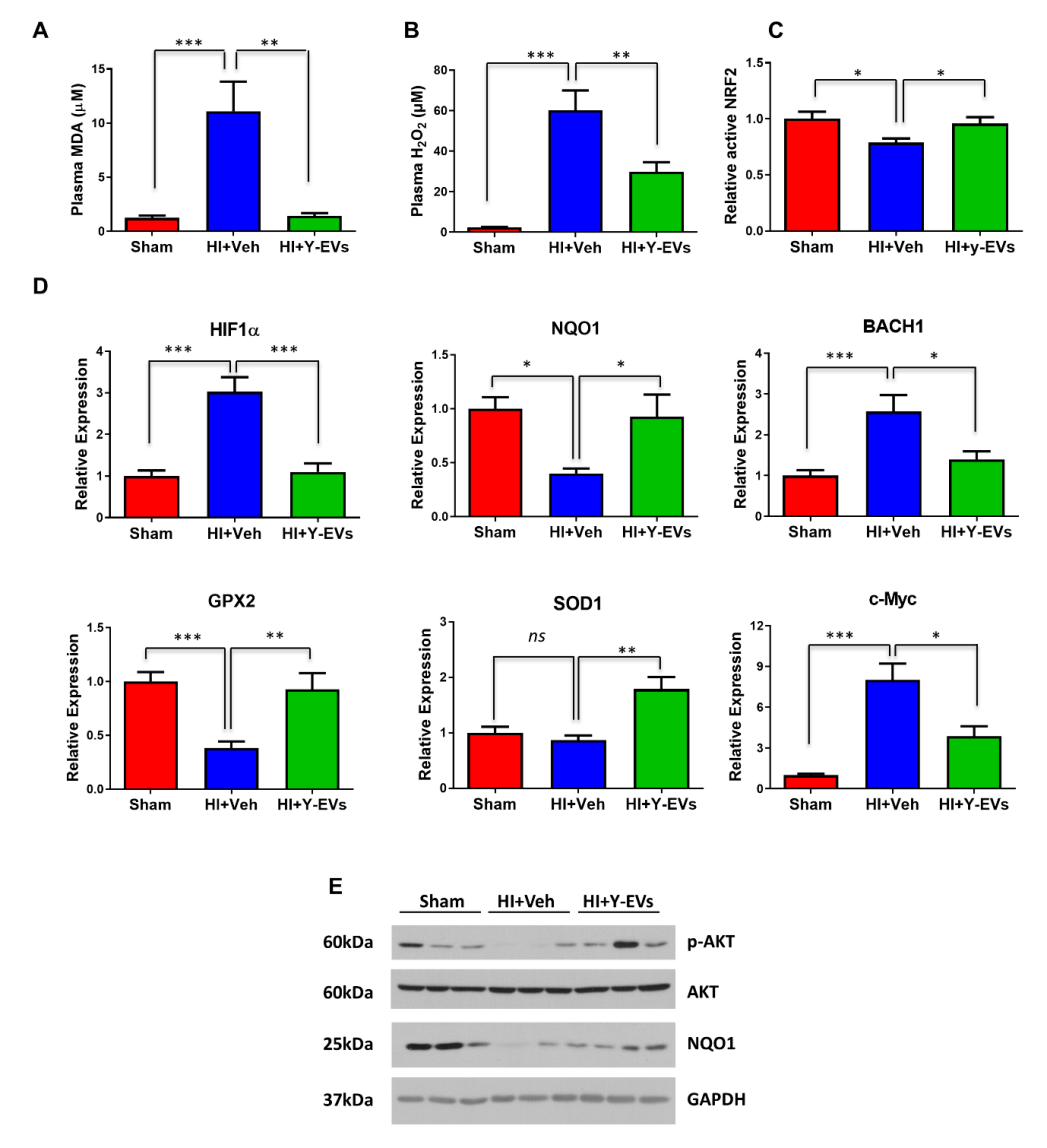
Y-EVs treatment on oxidative stress and antioxidant response following HI. (A-B) MDA and H_2_O_2_ concentrations in the plasma. The results shown represent mean ± SEM. ** =p<0.01 and *** =p<0.001. (C) Nrf2 transcriptional activity in liver. Mice liver nuclear fraction was tested for Nrf2 specific transcription factor DNA binding activity as described in the Methods section. The results shown represent mean ± SEM. * indicates p<0.05. (D) SYBR green real-time PCR amplification of NQO1, HIF1-a, GPX2, BACH1, SOD1 and c-Myc from the liver tissue. Groups: sham, HI+Veh and HI+Y-EVs. The results shown represent mean ± SEM. * =p<0.05, ** =p<0.01 and *** =p<0.001. (E) Representative Immunoblotting p-AKT, AKT and NQO1 in sham, HI+Veh and HI+Y-EVs. GAPDH was used as the loading control.

EVs regulate the activity of multiple physiological pathways, including the oxidative stress response, which is a hallmark of HI [31, 32]. We tested MDA and hydrogen peroxide (H_2_O_2_), two important oxidative stress markers in mice plasma. The concentration of both plasma MDA and H_2_O_2_ was significantly elevated in HI+veh group, and Y-EVs treatment effectively attenuated the increase (Fig. 5A-B). So, we next examined the expression changes in oxidative stress-related genes in the liver in the three groups of animals. However, as shown in Figure 5D, the expression of NQO1 and GPX2 in the liver were significantly decreased in mice subjected to HI, and restored in mice treated with Y-EVs, when compared to the Veh treated group. As both NQO1 and GPX2 are Nrf2-dependent antioxidant response genes, to explore the role of Y-EVs in Nrf2 signaling pathways, mice liver nuclear proteins were isolated and tested for the Nrf2 transcriptional activity. The change in expression level observed for the DNA-binding Nrf2 (Fig. 5C) followed the same trend as NQO1 and GPX2. However, Hif1α and Bach1 gene expression was significantly elevated following HI and Y-EVs treatment attenuated the increase (Fig. 5D). Furthermore, though the expression level of Sod1 showed no difference between the sham and HI+veh group, Sod1 was significantly increased with Y-EVs treatment further indicating an elevated antioxidant response with Y-EVs (Fig. 5D). The expression changes in the genes for NQO1, HMOX1, GPX2 and NRF2 were also observed in the heart tissue (Supplementary Fig. 1). We also found that, consistent with increased Nrf2 activity upon Y-EVs treatment, Y-EVs increased the level of phospho-AKT, an upstream mediator of Nrf2 signaling (Fig. 5E). This data suggests that the failing Nrf2 response following HI is related to elevated levels of Bach1 and that this could be a reason for the lack of significant upregulation of the antioxidant response genes, NQO1 and GPX2.

**Figure 6.**
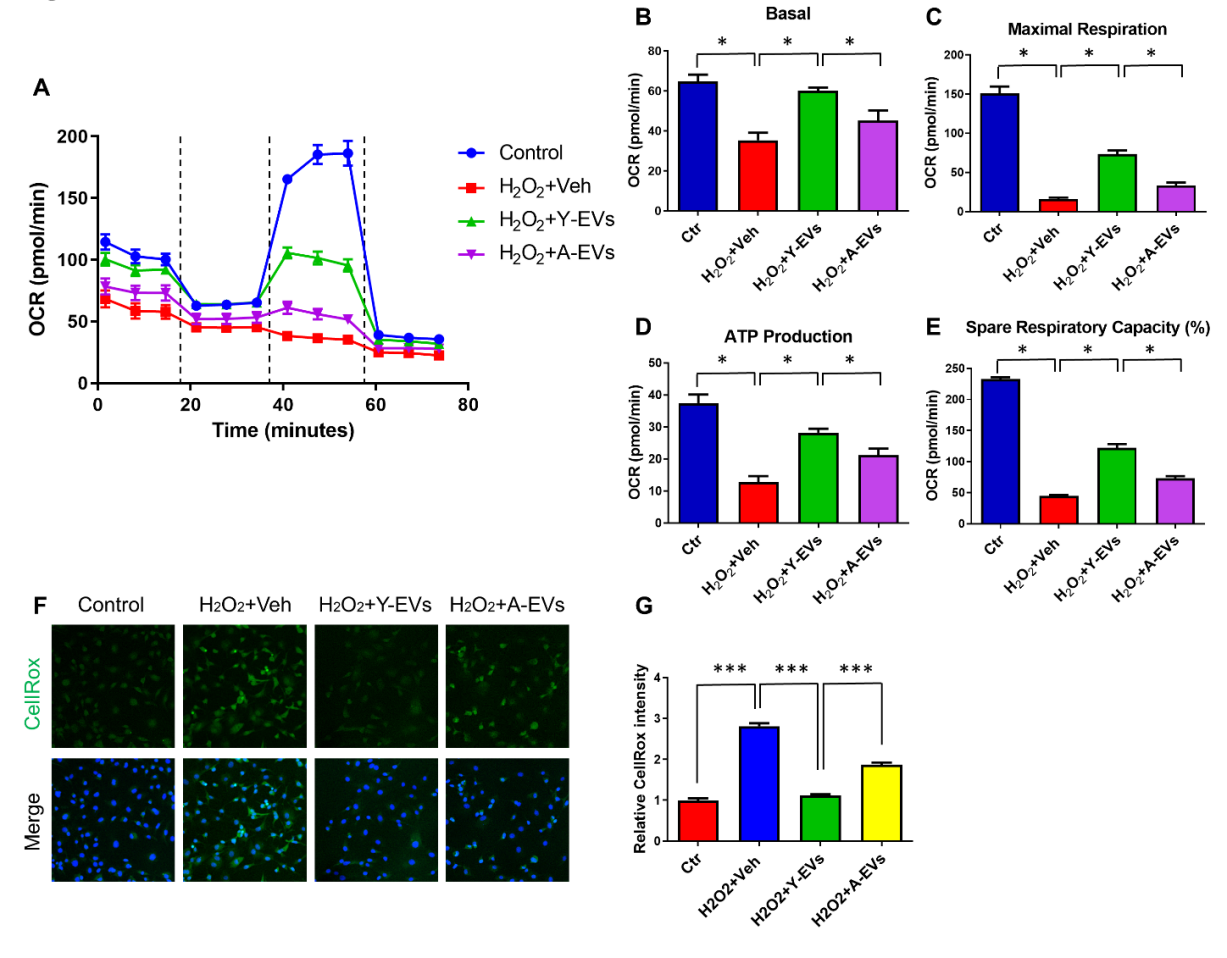
Effect of Y-EVs on mitochondrial function and oxidative stress induced in MEF cells. (A) Mitostress test with Y-EVs treated MEF cells following oxidative stress. MEF cells were treated with 200uM H_2_O_2_ and incubated with vehicle Y-EVs or A-EVs for 3 hours. Representative experiment. Mitostress test was performed by sequential addition of oligomycin (complex V inhibitor), FCCP (mitochondrial membrane depolarization), rotenone (complex I inhibitor) and antimycin (Anti A-complex III inhibitor). (B-E) Basal respiration, maximal respiration, ATP production, and spare respiration capacity were calculated from the data in panel A; * indicates p<0.05. (F) MEF cells were treated with 200uM H_2_O_2_, followed by vehicle, 100 µg/ml young EVs or aged EVs for 3 hours, then incubated with 5 μM CellROX® green reagent under culture conditions for 30 min and fixed with 4% PFA. Cells were stained with Hoechst for DNA. (G) CellROX green intensity was quantified using Image J software (NIH), *** indicates p < 0.001. At least 25 cells were measured in each group.

To understand the mechanisms underlying the effect of Y-EVs on HI associated oxidative stress, mouse embryonic fibroblast (MEF) cells were subjected to H_2_O_2_ -induced oxidative stress in an *in vitro* model and mitochondrial respiration was measured using the Seahorse extracellular flux (XF) analyzer. In this technique the mitochondrial oxygen consumption rate (OCR) is determined to measure oxidative phosphorylation (OXPHOS). H_2_O_2_ induced a decreased OCR demonstrating efficient reduction of mitochondrial respiration compared to the control (Fig. 6). This demonstrates significant impairment of the energetics process driven by H_2_O_2_-induced oxidative stress. However, Y-EVs restored OCR thereby rescuing (Fig. 6A-E) MEF cells from the severe oxidative stress induced by exposure to H_2_O_2_. To further confirm H_2_O_2_-induced oxidative stress, the cells were treated with veh, Y-EVs or A-EVs following H_2_O_2_ treatment, then stained with CellRox green. As shown in Figure 6F-G, H_2_O_2_ exposure significantly upregulated ROS production and Y-EVs treatment effectively attenuated the upregulation of ROS generation in response to H_2_O_2_. The oxidative stress induced in the presence of A-EVs was significantly higher than that observed in the presence of Y-EVs further demonstrating the antioxidant effect of Y-EVs.

**Figure 7.**
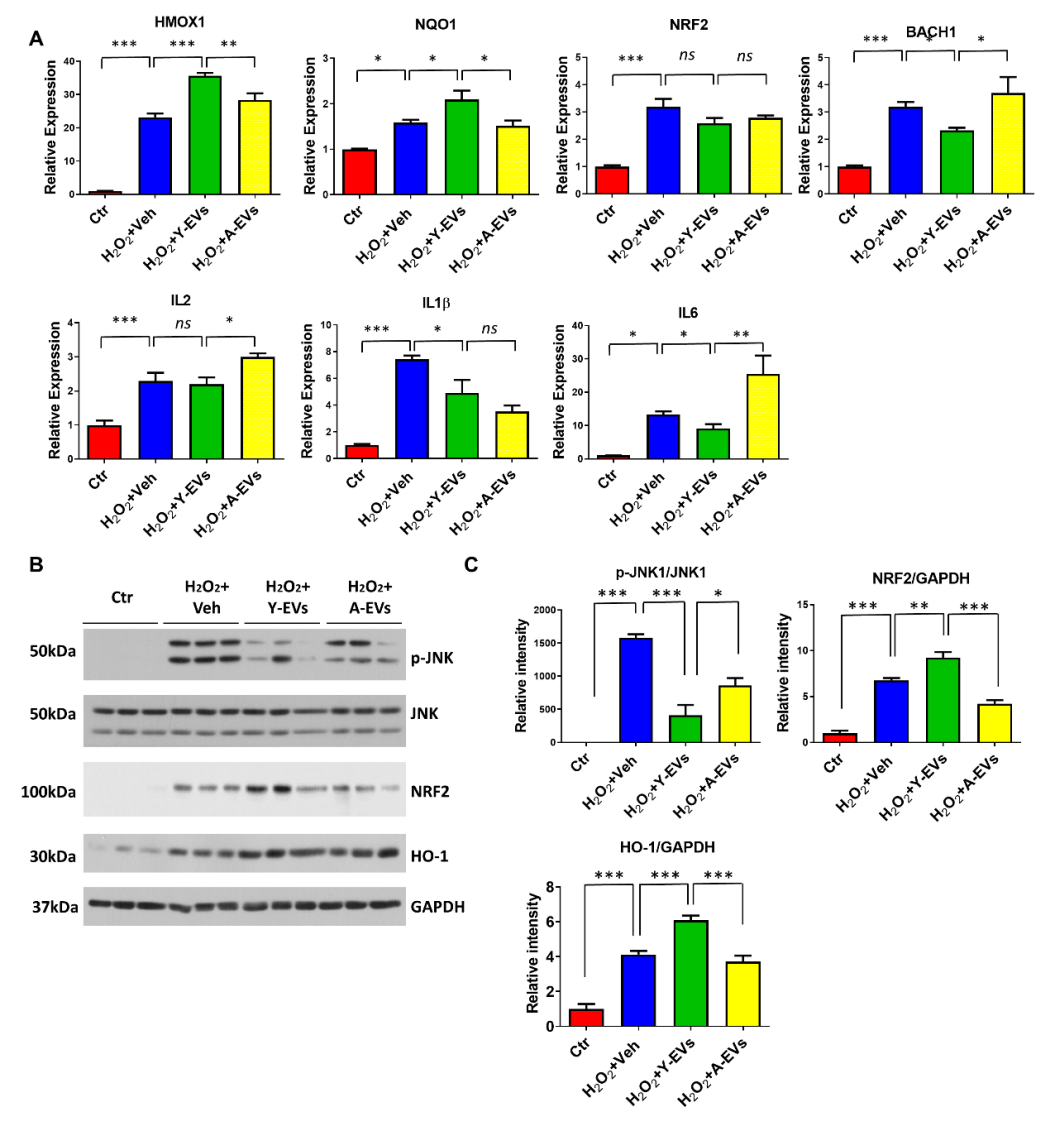
Effect of Y-EVs on oxidative stress and inflammation in MEF cells. (A) SYBR green real-time PCR amplification of HMOX1, NQO1, NRF2, BACH1, IL6, IL2 and IL1β. MEF cells were treated with 200uM H_2_O_2_, and added vehicle, Y-EVs or A-EVs for 3 hours. The results shown represent mean ± SEM. * indicates p<0.05; ns = not statistically significant. (B) Representative immunoblotting of p-JNK, JNK, NRF2 and HO-1 in control, H_2_O_2_+Veh, H_2_O_2_+Y-EVs and H_2_O_2_+A-EVs. MEF cells were treated with 200uM H_2_O_2_ and incubated with vehicle, Y-EVs or A-EVs for 3 hours. GAPDH was used as the loading control. (C) p-JNK/JNK, NRF2/GAPDH and HO-1/GAPDH ratios were quantified using Image J software (NIH), * indicates p < 0.05.

Next, we tested whether Y-EVs treatment could lead to modulation of Nrf2 signaling in H_2_O_2_ -induced oxidative stress *in vitro*, MEF cells were treated with vehicle, Y-EVs or A-EVs following H_2_O_2_ stress. The premise was based on our *in vivo* experiments that showed that intravenous administration of Y-EVs following HI led to the activation of Nrf2 signaling pathways and inhibition of oxidative stress in the mouse liver. The gene and protein expression of Nrf2 associated molecular mediators was tested as shown in Figure 7A, H_2_O_2_ treatment resulted in a significant increase in the expression of genes for Hmox1, NQO1, Nrf2 and Bach1. The data show an oxidative stress-driven antioxidant response with the H_2_O_2_ exposure. Whereas, Y-EVs treatment further increased the expression of Hmox1 and NQO1, Bach1 expression was reduced compared to that in veh or A-EVs treated cells. It was noteworthy that the treatment with veh, Y-EVs or A-EVs did not alter Nrf2 gene expression (Fig. 7A). As oxidative stress induces proinflammatory cytokine responses, we also checked the expression level of IL6, IL2 and IL1β. As shown in Figure 7A, H_2_O_2_ treatment promoted the expression of IL6, IL2 and IL1β, but treatment with Y-EVs significantly attenuated the expression of IL6 and IL1β, but not IL2, compared to veh treatment. Interestingly, A-EVs treatment further augmented the expression of IL6 and IL2 compared to that in Y-EVs treated cells (Fig. 7A). Whereas the in vitro experiments further demonstrate that Y-EVs reduce oxidative stress, our results also show that A-EVs cargo may contain factors that promote inflammation (Fig. 7).

**Figure 8.**
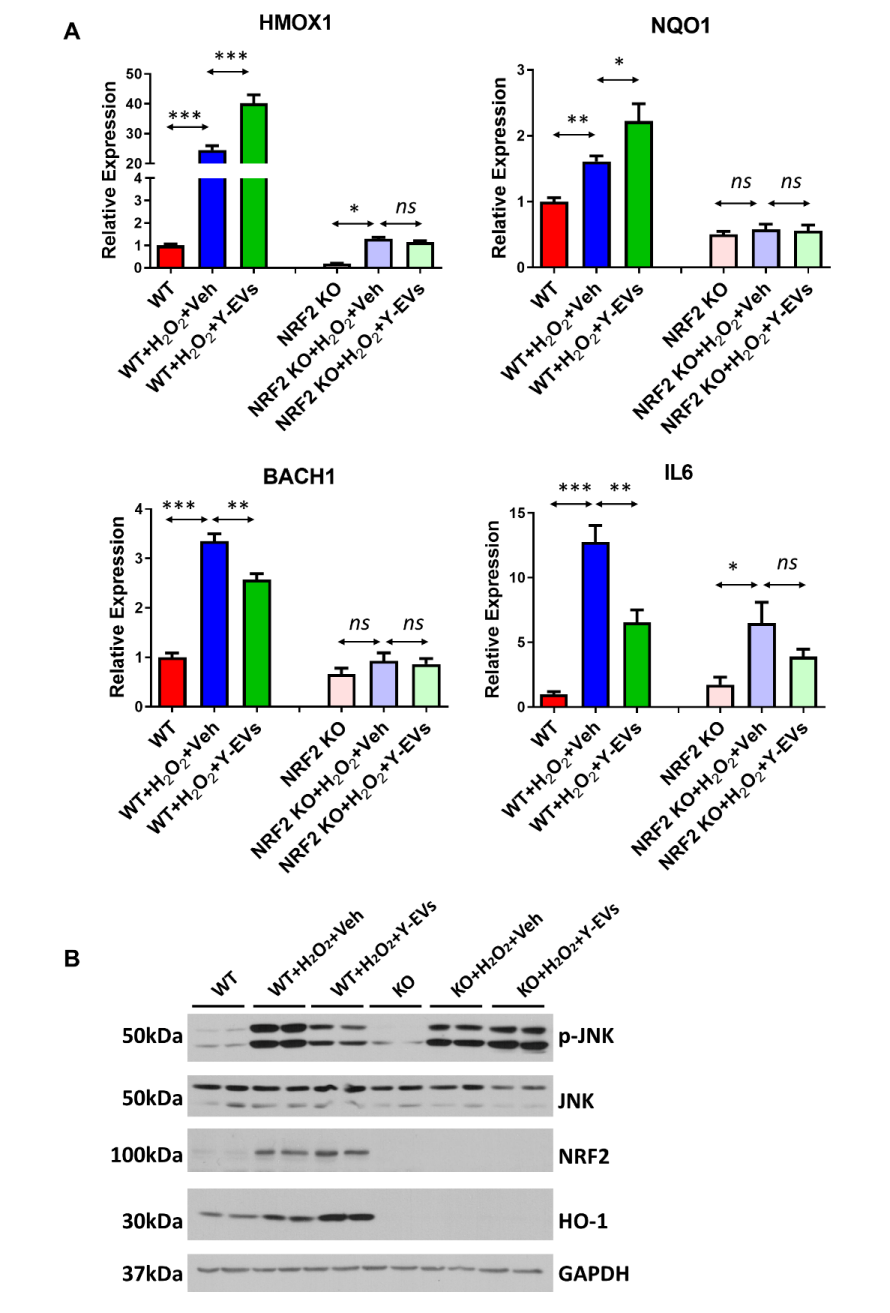
Effect of Y-EVs on MEF cells is Nrf2 dependent. (A) WT and NRF2 knock-out (KO) MEF cells were treated with 200uM H_2_O_2_, followed by vehicle or Y-EVs for 3 hours. SYBR green real-time PCR amplification of HMOX1, NQO1, BACH1 and IL6. The results shown represent mean ± SEM. * indicates p<0.05; ns = not statistically significant. (B) Representative Immunoblotting of p-JNK, JNK, NRF2 and HO-1 in control, H_2_O_2_+Veh, H_2_O_2_+Y-EVs and H_2_O_2_+A-EVs. WT and NRF2 knock-out MEF cells were treated with 200 µM H_2_O_2_, followed by vehicle, Y-EVs or A-EVs for 3 hours. GAPDH was used as the loading control. The blots shown are representative of the replicates.

To further determine the role of Y-EVs in Nrf2 signaling pathway, WT and Nrf2 knock out (KO) MEF cells were treated with veh or Y-EVs following H_2_O_2_ exposure. The phosphorylation of JNK, an injury marker, and the gene expression of Nrf2 associated genes were tested in cell lysates. The protein analysis showed Y-EVs treatment reduces the phosphorylation of JNK, an injury marker, following H_2_O_2_ treatment in WT cells. However, Y-EVs and Veh treatment following H_2_O_2_ exposure had a similar effect in Nrf2 KO cells (Fig. 8B). Not surprisingly, the levels of Hmox1 and NQO1 were increased while Bach1 and IL6 expression levels were decreased in Y-EVs treated WT MEF cells compared to Veh control. However, there was no significant difference in NQO1, Bach1 or IL6 in Nrf2 KO MEF cells with Y-EVs or Veh treatment (Fig. 8A). Nrf2 insufficiency had a negative impact on the ability of Y-EVs in protecting MEF cells from oxidative stress establishing that Bach1/Nrf2 axis as a critical target of Y-EVs.

## DISCUSSION

Plasma EVs contain a heterogeneous group of macro and micro molecules derived from a variety of organismal cells. The cargo of the EVs derived from a normal organism may vary depending on age, sex, genetic background, and the environment. Blood from young animals has been known to have a reparative effect in aging and disease, though the function and identity of the molecular species responsible for the salutary effect remain elusive [33]. Our experiments show that EVs in the blood of young mice have a reparative effect in HI and that the reparative effect observed is due to one or more maturational factors, as the resilience seen in the young mice disappeared in adulthood. Furthermore, the studies demonstrate that the juvenile plasma factors alleviate oxidative stress and improve organ function in the liver. The liver is among the first and major organs affected by the hypoxic insult due hemorrhagic shock [27]. The oxidative injury caused by resuscitation also affects the liver [34]. Though our data suggest that Y-EVs treatment can enhance antioxidant responses and reduce or alleviate oxidative stress following HI, the deficiency of a robust antioxidant response following HI as evidenced by the decreased expression of NQO1, GPX2 and nuclear Nrf2 was surprising. The lack of an increase in the expression of these genes in response to HI-induced oxidative stress is likely a Bach1-mediated effect, a Bach1-dependent impaired Nrf2 inducibility was previously observed with aging [35]. While an increase in the expression of HIF-1α following HI was expected due to HI-induced tissue hypoxia, an increased Bach1 expression after HI was suggestive of Nrf2 dysregulation in HI [36]. Bach1 competes for the Nrf2-MAF binding site on antioxidant response elements (ARE) on Nrf2 target genes and negatively regulates Nrf2 function [37, 38]. Our results show that the maturation-dependent factors in the Y-EVs enhance antioxidant response by regulating the Bach1-Nrf2 axis.

We also found a significant reduction in the expression of phosphorylated JNK, phosphorylated eIF2α, as well as NLRP3 in Y-EVs treated animals as compared to vehicle treated animals subjected to HI. JNK/NLRP3 inflammatory signaling plays an important role in mediating hemorrhagic shock-induced tissue injury and JNK is activated by hypoxia, ROS or RNS, and/or various cytokines [39-41]. Moreover, c-Jun/AP-1 activation was associated with an increased inflammatory response, apoptosis, and tissue injury after both H/R and hypoxia. The inhibition of JNK by the protease-resistant JNK inhibitor (D-JNKI-1) before the onset of hemorrhage blunted hepatic damage and local and systemic inflammatory changes after H/R[42]. JNK inhibition after post-hemorrhagic shock also attenuated H/R-induced hepatic injury, neutrophil infiltration, and the production and release of proinflammatory cytokines [43]. The attenuation of the JNK/NLRP3 mediated inflammation following Y-EVs treatment after HI may be attributed to the Nrf2 mediated antioxidant response.

Though it has been recognized for a long time that factors in the blood of young organisms can rejuvenate the old ones, the nature and function of these maturational factors were not fully understood [44-46]. Recent studies using heterochronic parabiosis models further reinforced the hypothesis that juvenile factors can rejuvenate aged systems [47]. A recent study by Rando and colleagues demonstrated a decline of regeneration potential with age that could be reversed through systemic factors in the young [19]. Another study using heterochronic parabiosis showed that old blood has the potential to reduce mitochondrial content and the levels of enzymes involved in oxidative phosphorylation suggesting that age-associated changes in mitochondrial function is influenced by circulating factors [48]. It will be interesting to know whether the EVs isolated from aged mice could aggravate outcome following HI in young mice, as well as their effect on mitochondrial function. Nevertheless, emerging studies indicate an important role for biological fluid-derived EVs including exosomes in cell-cell communications and repair [49-51]. Paracrine signaling as well as distant signaling by soluble factors from stem cells are being increasingly recognized as relevant to disease treatment [52-54]. An increased tissue ATP level, declined oxidative stress and reduced infarct size were observed, in another study, when a single intravenous dose (4 mg/Kg) of stem cell-derived exosomes was given prior to myocardial I/R injury [55].

An empirical analysis of the cargo of Y-EVs to determine the maturational factor is challenging due to the heterogeneous nature of the constituent molecular species. Nevertheless, such unknown systemic factors are postulated to mediate effects on multi-organ function and survival in individuals exposed to insults, and these effects are age and maturational dependent [56-58]

The identification of endogenous juvenile protective factors that demonstrate a salutary effect on aging may not only mitigate the influence of aging on the injury, but may also enable injury resolution and will open a new dimension in the treatment of injuries such as that due to severe blood loss. There is also a need, albeit challenging, to further define the mechanistic underpinnings that confer a protective effect in hemorrhagic shock with respect to maturation and aging including their participation in organ crosstalk and long-distance information sharing. Our finding of the resilience of youthful organisms in response to injury, the salutary effect of plasma-derived EVs from the young in altering outcomes following hemorrhagic shock, and their ability to modulate antioxidant response in the aged animal have profound implications not only for the treatment of low flow conditions, but also for the understanding of the biology of aging.

## Supplementary Materials

The Supplementary data can be found online at: www.aginganddisease.org/EN/10.14336/AD.2021.0830.


