# Supplementary material for: Juvenile Plasma Factors Improve Organ Function and Survival following Injury by Promoting Antioxidant Response
Source: Aging Dis. 2022 Apr 1;13(2):568–82. doi: 10.14336/AD.2021.0830 (PMC8947827; doi:10.14336/AD.2021.0830)
Supplement: Supplementary file 1 [file AD-13-2-568-s.pdf]

SUPPLEMENTARY DATA

Juvenile Plasma Factors Improve Organ Function and Survival following Injury by Promoting Antioxidant Response

Xiaogang Chu[1], Kumar Subramani[1], Bobby Thomas[2], Alvin V. Terry, Jr.[1], Sadanand Fulzele[3], Raghavan Pillai Raju[1,*]

© 2021. Chu X et al. Published online at http://www.aginganddisease.org/EN/10.14336/AD.2021.0830

SUPPLEMENTARY DATA

Supplementary Table 1. Antibodies used.

Antibodies for Western Blot	Host species	Dilution	Source	Catalogue
Phospho-SAPK/JNK (Thr183/Tyr185)	Rabbit	1:1000	Cell Signaling	4668
SAPK/JNK	Rabbit	1:1000	Cell Signaling	9252
Phospho-eIF2α (Ser51)	Rabbit	1:1000	Cell Signaling	3398
eIF2α	Rabbit	1:1000	Cell Signaling	5324
Phospho-p38 MAPK (Thr180/Tyr182)	Rabbit	1:1000	Cell Signaling	4511
p38 MAPK Antibody	Rabbit	1:1000	Cell Signaling	9212
Phospho-Akt (Ser473)	Rabbit	1:1000	Cell Signaling	4060
Akt (pan)	Rabbit	1:1000	Cell Signaling	4691
GAPDH	Rabbit	1:5000	Cell Signaling	2118
NRF2	Rabbit	1:1000	Proteintech	16396-1-AP
NLRP3	Mouse	1:3000	Adipogen	A27381510
Anti-Heme-Oxygenase-1	Rabbit	1:1000	Stressgen	SPA-896
NQO1	Rabbit	1:1000	Abcam	ab34173

Supplementary Table 2. Primer sequences.

Gene	Forward primer sequence (5'-3')	Reverse primer sequence (5'-3')
IL6	TGGAGTCACAGAAGGAGTGGCTAAG	TCTGACCACAGTGAGGAATGTCCAC
IL1β	CCCTGCAGCTGGAGAGTGTGG	TGTGCTCTGCTTGAGAGGTGCT
IL10	TGCGACGCTGTCATCGATTT	GTAGATGCCGGGTGGTTCAA
IL2	AACCTGAAACTCCCCAGGAT	CGCAGAGGTCCAAGTTCATC
Tnf-a	ATAGCTCCCAGAAAAGCAAGC	CACCCCGAAGTTCAGTAGACA
HMOX1	GGTGATGGCTTCCTTGTACC	AGTGAGGCCCATACCAGAAG
NQO1	TTCTGTGGCTTCCAGGTCT T	AGGCTGCTTGGAGCAAAAT
GPX2	ATCAAACGGCTCCTCAAAGT	GGGACGATATTCAGGGAATG
SOD1	CGGATGAAGAGAGGCATGTT	CACCTTTGCCCAAGTCATCT
HIF-1α	AGCCCTAGATGGCTTTGTGA	TATCGAGGCTGTGTCGACTG
c-Myc	AAAGGCCCCCAAGGTAGTTA	CTCGCCGTTTCCTCAGTAAG
BACH1	GCGCAGAGGGAGTGAGTC	GGGAGAGCAGTGGAAAGCAT
NRF2	TTTGTAGATGACCATGAGTCGC	TCCTGCCAAACTTGCTCCAT
β-actin	CGCCACCAGTTCGCCATGG	TACAGCCCGGGGAGCATCG

© 2021. Chu X et al. Published online at http://www.aginganddisease.org/EN/10.14336/AD.2021.0830

SUPPLEMENTARY DATA

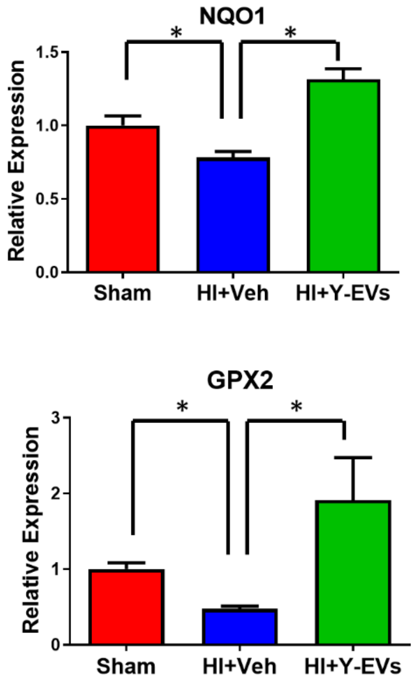

Supplementary Figure 1: SYBR green real-time PCR amplification of HMOX1, NQO1, GPX2 and NRF2 in sham, HI+Veh and HI+Y-EVs mouse heart tissues. The results represent mean ± SEM for six animals in each group. * indicates $p<0.05$,

© 2021. Chu X et al. Published online at http://www.aginganddisease.org/EN/10.14336/AD.2021.0830
